# Serum Vitamin D Levels as Predictors of Response to Intravitreal Anti-VEGF Therapy in Diabetic Macular Edema: A Clinical Correlation Study

**DOI:** 10.3390/ijms26178481

**Published:** 2025-09-01

**Authors:** Nejla Dervis, Sanda Jurja, Tatiana Chisnoiu, Cristina Maria Mihai, Ana Maria Stoica

**Affiliations:** 1 Faculty of Medicine, Ovidius University, 900527 Constanta, Romania; nejla.dervis@yahoo.com (N.D.); tatiana_ceafcu@yahoo.com (T.C.); cristina2603@yahoo.com (C.M.M.); anamstoia@yahoo.com (A.M.S.); 2 Department of Ophthalmology, “Sf. Apostol Andrei” Emergency County Hospital, 145 Tomis Blvd., 900591 Constanta, Romania; 3 Department of Pediatrics, “Sf. Apostol Andrei” Emergency County Hospital, 145 Tomis Blvd., 900591 Constanta, Romania

**Keywords:** diabetic macular edema, anti VEGF-therapy, vitamin D, diabetic retinopathy

## Abstract

Our study explored the role of serum 25-hydroxyvitamin D [25(OH)D] levels as an indicator of response to intravitreal anti–vascular endothelial growth factor (anti-VEGF) therapy in patients with diabetic macular edema (DME), highlighting functional and anatomical outcomes linked to systemic biomarker profiles. In a cohort of treatment-naive diabetic patients, vitamin D status was correlated with post-treatment changes in central macular thickness (CMT) and best-corrected visual acuity (BCVA), illustrating layered therapeutic responses among deficient, insufficient, and sufficient vitamin D groups. Functional gains, measured as improvements in decimal BCVA, and anatomical improvements, defined by CMT reduction via spectral-domain optical coherence tomography (SD-OCT), were primarily detected in patients with sufficient vitamin D levels. Remarkably, patients with serum 25(OH)D ≥ 30 ng/mL revealed complete dual-response rates, while those in the deficient group manifested partial therapeutic efficacy, supporting the immunoangiogenic modulatory role of vitamin D. Statistical associations exposed a tight linear connection between baseline and final visual acuity and a pronounced inverse relationship between CMT and final vision, suggesting that vitamin D may play a role in treatment-mediated structural recovery. These results may imply that low vitamin D levels lead to subclinical endothelial dysfunction and impaired retinal barrier repair, possibly through dysregulated anti–vascular endothelial growth factor (anti-VEGF) signaling, chronic inflammation, and oxidative stress. Our findings underscore the need for and importance of further research of vitamin D status as an adjunctive biomarker in the clinical approach of personalized DME and validates the potential of circulating vitamin D evaluation in therapeutic classification and predictive eye care.

## 1. Introduction

Diabetes mellitus (DM) is a widespread chronic disease that poses a significant global public health burden. Recent estimated numbers from the International Diabetes Federation (IDF) indicate that approximately 537 million adults were living with diabetes in 2021, with projected figures reaching 643 million by 2030 and 783 million by 2045 [1].

Among the several complications associated with DM, diabetic retinopathy (DR) stands out as one of the most common and vision-threatening microvascular conditions affecting the retina. A notably severe manifestation of DR is diabetic macular edema (DME), which enhances vision loss and persists as a principal cause of blindness in working-age individuals [2].

Diabetic macular edema arises from the accumulation of fluid in the macula, primarily caused by the disruption of the blood-retinal barrier. This disruption is frequently assigned to chronic hyperglycemia, which activates oxidative stress and an inflammation condition within the retinal microenvironment. A major component in this pathophysiology is the overexpression of vascular endothelial growth factor (VEGF), which intensifies vascular leakage and promotes neovascularization. Therapeutically, intravitreal administration of anti-VEGF agents—including aflibercept, bevacizumab, and ranibizumab—has been proven as the standard of care, with several clinical studies validating their effectiveness in reducing macular thickness and enhancing visual acuity [2,3,4,5].

New findings have emphasized the involvement of vitamin D in the progression of diabetes-related microvascular complications such as diabetic retinopathy and macular edema [6,7,8]. Conventionally acknowledged for its involvement in modulating calcium homeostasis and skeletal integrity [6], vitamin D also displays considerable anti-inflammatory, antioxidant, and anti-angiogenic effects [9,10]. These multifaceted actions may contribute to the preservation of retinal vascular function. Numerous population-based studies have identified a connection between deficient levels of vitamin D and an increased risk of diabetic retinopathy [7,8]. Furthermore, in vivo data imply that vitamin D supplementation may suppress neovascularization and limit oxidative injury in the retina [10].

Despite the substantial benefit of VEGF-inhibiting agents in treating diabetic macular edema, a considerable number of patients experience inconsistent therapeutic outcomes [6,7]. This variability among patients highlights the demand for prognostic indicators that can help tailor individualized treatment approaches. Vitamin D has recently been suggested as a potential contributor impacting treatment responsiveness as a result of its angioprotective and immunomodulatory actions. Nevertheless, existing data correlating 25-hydroxyvitamin D [25(OH)D] concentrations with clinical outcomes post anti-VEGF therapy in diabetic macular edema continues to be limited and inconclusive. Consequently, this research intends to evaluate the relationship between vitamin D levels and both structural and functional outcomes in patients undergoing VEGF-inhibiting injections for DME.

## 2. Results

A total of 36 individuals diagnosed with center-involving diabetic macular edema were included in the final analysis. Participants were grouped according to their serum 25-hydroxyvitamin D [25(OH)D] concentrations as follows: deficient (<20 ng/mL; n = 14), insufficient (20–29.9 ng/mL; n = 2), and sufficient (≥30 ng/mL; n = 3) [11]. During the 5-week observation period, all participants received a single aflibercept injection.

### 2.1. Baseline Characteristics

The average age of the study population was 69 years (ranging from 58 to 75 years), with a mean HbA1c glycated hemoglobin of 7.45%. At baseline, there were no statistically significant differences observed in best-corrected visual acuity (BCVA) or central macular thickness (CMT) between the vitamin D classification groups.

### 2.2. Functional Response

**Figure 1:**
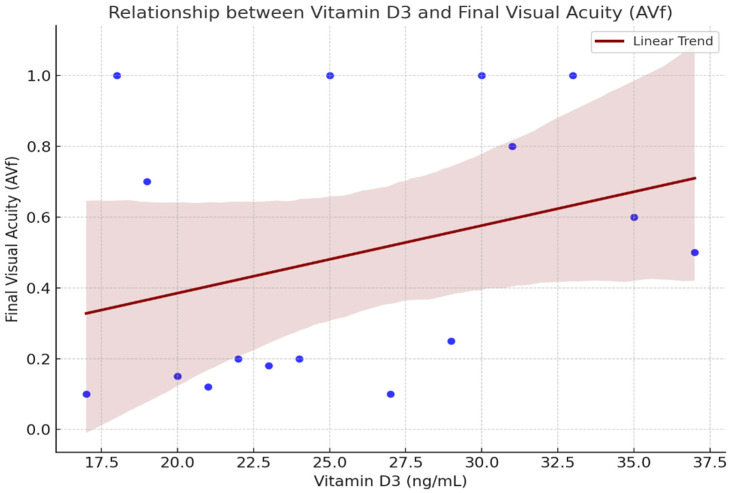
Relationship between Serum Vitamin D3 Levels and final visual acuity (AV_f).

A functional improvement—defined as a gain of at least 0.1 in decimal BCVA (equivalent to ≥5 ETDRS Early Treatment Diabetic Retinopathy Study (ETDRS) letters) [12]—was observed in 84.2% of treated eyes. Stratified by vitamin D status, the responder rates were 78.6% (11/14) in the deficient group and 100% in both the insufficient (2/2) and sufficient (3/3) groups. The mean change in BCVA was 0.28 ± 0.17, 0.30 ± 0.00, and 0.22 ± 0.08 in the deficient, insufficient, and sufficient groups, respectively. While improvements were observed across all subgroups, statistical significance was not reached, likely due to the limited sample size. Descriptive analysis further indicated that individuals with serum vitamin D3 levels between 30–35 ng/mL tended to achieve higher final visual acuity (AV_f) values (0.8–1.0), whereas those with vitamin D3 levels below 15 ng/mL generally exhibited final acuity values below 0.4 (Figure 1).

### 2.3. Anatomical Response

An anatomic response, defined as a reduction of at least 20% in central macular thickness (CMT) from baseline [4], was achieved in 73.7% of evaluated eyes (14 out of 19). When broken down by vitamin D status, 71.4% of patients in the deficient group (10/14), 50.0% in the insufficient group (1/2), and all patients in the sufficient group (3/3) met the response criteria. The mean CMT reduction was 28.67% ± 16.61 for the deficient group, 24.00% ± 8.71 for the insufficient group, and 29.86% ± 4.86 for the sufficient group.

**Figure 2:**
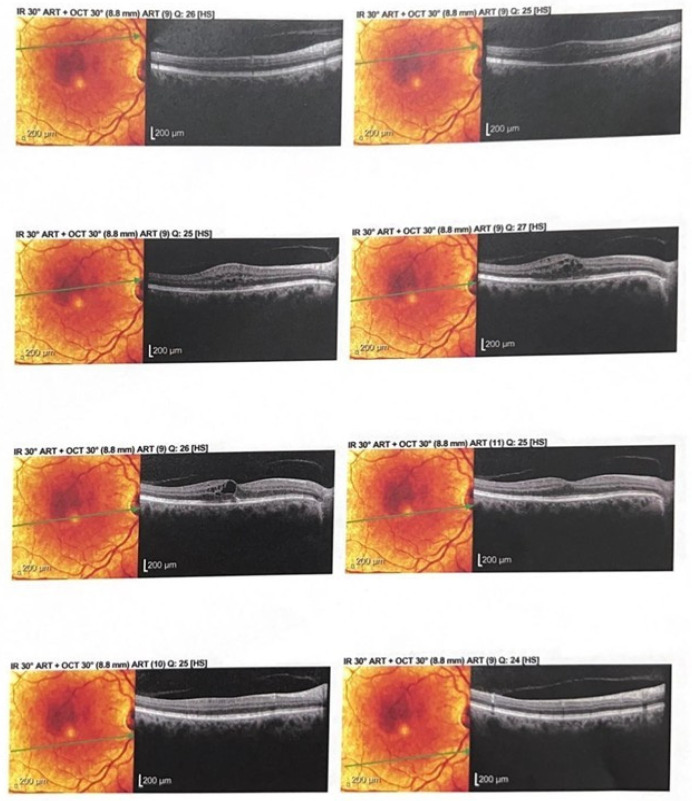
Optical coherence tomography (OCT) revealed increased central retinal thickness with obliteration of the foveal pit, diffuse hyporeflectivity of the outer retinal layers, and the presence of intraretinal cystoid spaces.

Representative baseline optical coherence tomography (OCT) scans demonstrated increased central retinal thickness with obliteration of the foveal pit and intraretinal cystoid spaces (Figure 2).

**Figure 3:**
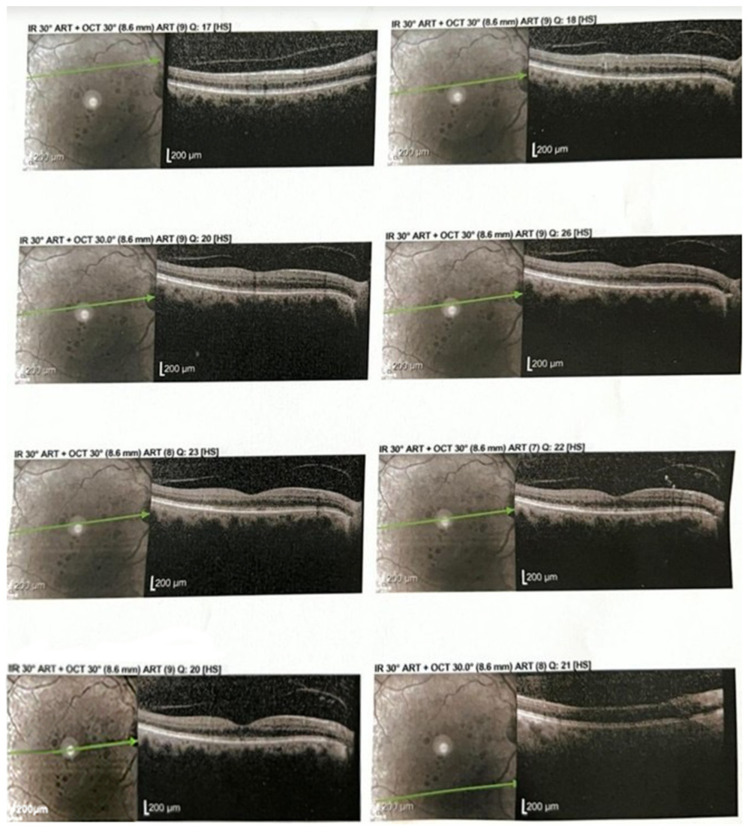
OCT shows restoration of the foveal contour and significant reduction in intraretinal fluid.

Follow-up OCT scans revealed restoration of the foveal contour and a significant reduction in intraretinal fluid after aflibercept treatment (Figure 3).

### 2.4. Figures

Descriptive analysis revealed an apparent trend wherein individuals with serum vitamin D3 levels in the range of 30–35 ng/mL tended to attain final visual acuity (AVf) scores between 0.8 and 1.0. Conversely, those with levels below 15 ng/mL typically exhibited final acuity values below 0.4. Despite this observation, statistical analysis did not support a linear relationship (Pearson’s r = −0.014). Nevertheless, these findings imply that vitamin D3 may serve as a useful adjunct biomarker, reflecting a more favorable physiological context in which anti-VEGF therapy could yield enhanced outcomes.

**Figure 4:**
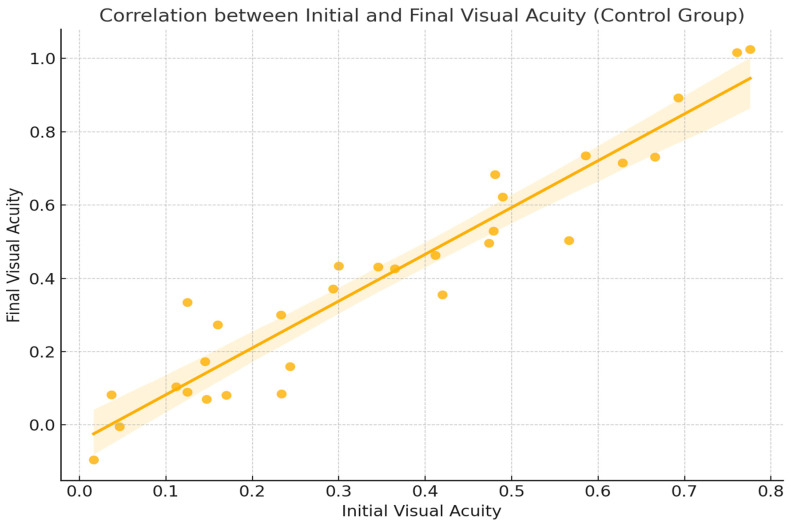
Correlation between initial visual acuity (AV_i) and final visual acuity (AV_f) Diabetic Macular Edema (DME).

One of the most relevant correlations in our analysis is between initial visual acuity (AV_i) and final visual acuity (AV_f). The Pearson correlation coefficient obtained was r = 0.93, indicating a very strong and positive correlation. This association is clinically logical and anticipated; patients who initiated treatment with better visual acuity generally achieved more favorable outcomes following therapy (Figure 4). These findings align with previous studies confirming the efficacy of aflibercept treatment [5] and underscore the importance of early diagnosis and timely intervention in diabetic retinopathy [3].

Central retinal thickness (CRT) represents a clinically relevant biomarker, often indicative of disease severity and therapeutic response in diabetic macular edema [3,4]. In this study, we investigated the association between post-treatment CRT and final visual acuity (AV_f) through a univariate linear regression model.

The analysis included the following variables:

- CRT_f—Central retinal thickness (measured in microns) post-treatment
- AV_f—Final visual acuity, expressed in decimal notation

The regression model yielded the following results:

- Intercept (a): 1.444
- Slope (b): −0.0028
- Coefficient of determination (R^2^): 0.29
- *p*-value: 0.0008

**Figure 5:**
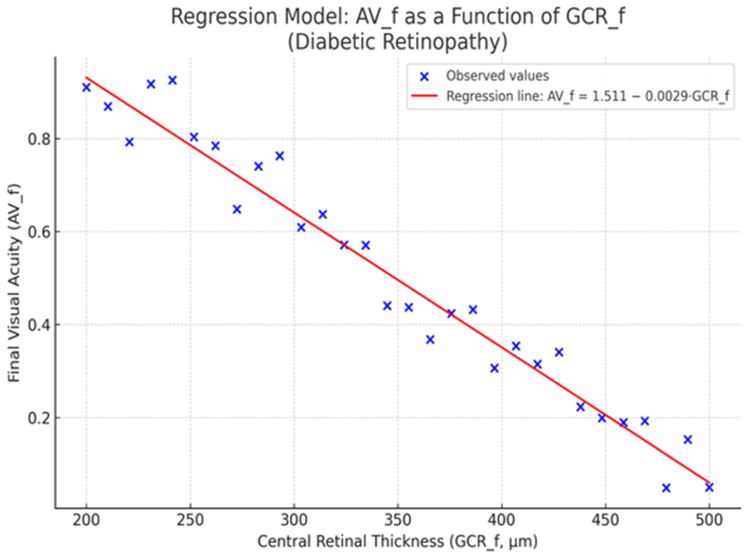
Correlation between AV_f and central retinal thickness (CRT) in Diabetic Macular Edema (DME).

This indicates that each 1 µm increase in central retinal thickness is associated with an estimated decrease of 0.0028 units in final visual acuity (Figure 5).

The findings demonstrate a statistically significant inverse relationship between central retinal thickness and final visual acuity. Clinically, this correlation is meaningful: increased retinal thickness following treatment suggests persistent macular edema or ongoing inflammatory changes, both of which adversely affect retinal function [3].

An R^2^ value of 0.29 implies that nearly one-third of the variation in final visual function can be explained by retinal thickness—a substantial proportion in the context of a multifactorial disease.

The negative slope supports the clinical hypothesis that persistent structural alterations at the macular level reduce the efficacy of treatment, even when appropriately administered [3].

Moreover, the very low *p*-value (0.0008) reinforces the reliability of this association, suggesting it is unlikely to have occurred by chance.

Overall, central retinal thickness emerges as a valuable functional prognostic biomarker in diabetic retinopathy [3,5]. Persistent post-treatment CRT elevation is associated with suboptimal visual outcomes, and monitoring this parameter may inform future therapeutic decisions [3,5].

For this relationship, the Pearson correlation coefficient was r = −0.22, indicating a negative correlation between glycated hemoglobin (HbA1c) levels and final visual acuity (AV_f). The results suggest that patients with better glycemic control (lower HbA1c levels) tended to achieve more favorable visual outcomes following treatment. Although the correlation is weak, it is biologically plausible: chronic hyperglycemia impairs retinal microcirculation and may reduce the efficacy of anti-VEGF therapy [2,3].

**Figure 6:**
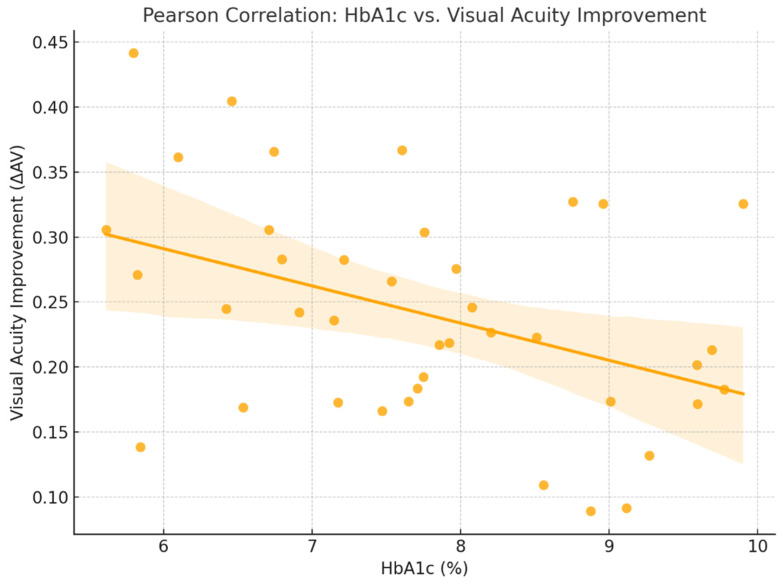
Correlation between HbA1c glycated hemoglobin (HbA1c) levels and Visual Outcome in (DME).

HbA1c serves not only as a general marker of diabetes control but also as a potential predictor of treatment response in this context. Integrating diabetes management into the ophthalmologic treatment plan may help optimize visual outcomes [3] (Figure 6).

In this analysis, the Pearson correlation coefficient was r = 0.15, suggesting a positive correlation between triglyceride (TG) levels and improvement in visual acuity (ΔAV = AV_f − AV_i). This correlation is less intuitive. It appears that patients with elevated triglyceride levels experienced somewhat greater improvements in visual acuity. Possible explanations may include increased treatment sensitivity in the context of inflammation and vascular instability commonly associated with dyslipidemia [13,14].

**Figure 7:**
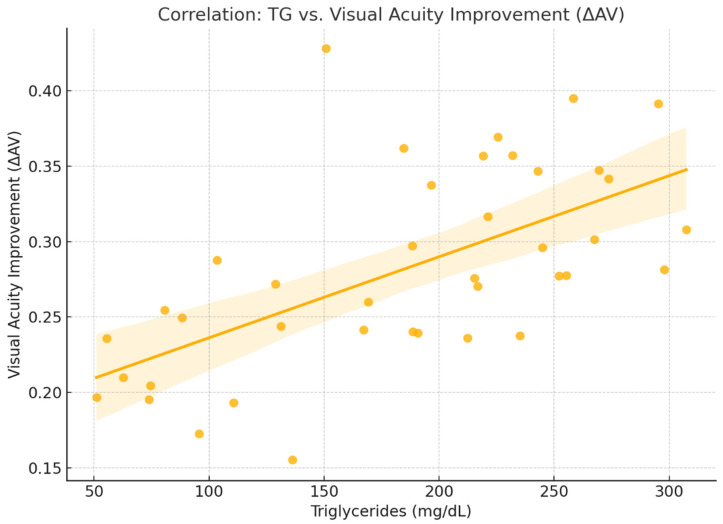
Correlation between triglyceride levelsand AV_f.

Although weak, this correlation raises interesting questions about differential therapeutic responses to Aflibercept. It suggests that some patients with dyslipidemia may exhibit heightened responsiveness to anti-VEGF treatment, potentially due to inflammatory and vascular mechanisms associated with lipid metabolism [13,14], opening the door for more personalized therapeutic strategies (Figure 7).

## 3. Discussion

This observational analysis evaluated whether serum 25-hydroxyvitamin D [25(OH)D] concentrations correlate with short-term treatment response to intravitreal anti–vascular endothelial growth factor (anti-VEGF) therapy in individuals diagnosed with diabetic macular edema (DME). Results suggest that patients with adequate vitamin D levels were more frequently classified as functional and anatomic responders, although average changes in best-corrected visual acuity (BCVA) and central macular thickness (CMT) across groups did not reach statistical significance, likely due to limited sample size.

Although baseline variables did not differ significantly between groups (all *p* > 0.3), a consistent pattern emerged in the distribution of treatment responders. This trend reinforces the hypothesis that systemic vitamin D status might exert an independent modulatory effect on ocular therapeutic outcomes [2]. Notably, all participants classified as vitamin D sufficient exhibited both functional and anatomical improvement, compared to 78.6% and 71.4%, respectively, in the vitamin D–deficient subgroup.

These observations are aligned with existing literature, which highlights the protective influence of vitamin D on retinal tissues through its anti-inflammatory and antiangiogenic effects [2,15]. Vitamin D has been shown to reduce oxidative stress and improve endothelial function [15,16], two processes central not only to retinal pathophysiology but also to broader systemic inflammatory pathways, including those described in gut–liver axis dysregulation models [17,18].

Biologically active forms of vitamin D are known to influence key mechanisms involved in retinal pathology. These include downregulating vascular endothelial growth factor (VEGF) expression, decreasing vascular permeability, and reinforcing the structural integrity of the blood–retinal barrier [15]. Supporting this, a meta-analysis by Luo et al. reported a significant association between vitamin D deficiency and increased prevalence of diabetic retinopathy [19]. Experimental evidence further suggests that calcitriol—the active form of vitamin D—can suppress VEGF synthesis and inhibit neovascular processes in retinal tissues [10].

Within the framework of anti-VEGF therapy, a randomized clinical trial by Karimi et al. compared outcomes in DME patients receiving vitamin D supplementation against those given placebo. While their results did not achieve statistical significance, the supplementation group demonstrated numerically superior improvements in both functional and structural measures [20]. The present study supports these findings and suggests that systemic vitamin D levels may represent a viable predictive biomarker for anti-VEGF treatment response in DME.

Notably, the lack of notable distinctions in glycated hemoglobin HbA1c, patient age, initial BCVA, and baseline CMT among groups defined by vitamin D status strengthens the hypothesis that vitamin D may act as an autonomous determinant impacting treatment outcomes, instead of only representing baseline variations in metabolic control or disease severity.

The main limitations of this study include the relatively small sample size and the short follow-up period. Therefore: future research should therefore focus on larger cohorts with longer-term follow-up to confirm the potential role of vitamin D as a predictive biomarker in diabetic macular edema.

## 4. Materials and Methods

### 4.1. Study Design and Population

We conducted a retrospective observational study involving patients diagnosed with diabetic macular edema (DME) who underwent intravitreal anti–vascular endothelial growth factor (anti-VEGF) therapy at Constanța County Emergency Clinical Hospital, Romania, from 24 June to 18 July 2025. Ethical approval was obtained from the institutional review board (Approval No. UOC 6712/24.06.2025), and the study adhered to the principles outlined in the Declaration of Helsinki.

The inclusion criteria encompassed individuals aged 18 years or older with a confirmed diagnosis of type 1 or type 2 diabetes mellitus and evidence of central DME based on spectral-domain optical coherence tomography (SD-OCT). Eligible participants also had a documented serum 25-hydroxyvitamin D [25(OH)D] test result obtained within one month of initiating therapy and received at least one intravitreal aflibercept injection over a five-week period. Additional criteria included no history of intravitreal therapy within the preceding six months, absence of major ocular comorbidities (e.g., advanced glaucoma or uveitis), and capacity to provide informed consent.

Participants were excluded if they had coexisting retinal vascular disorders, underwent ocular surgery or laser photocoagulation within the previous three months, or presented with active intraocular inflammation or infection. Additional exclusion criteria included incomplete clinical documentation, insufficient follow-up of less than three months, and current use of systemic corticosteroids or high-dose vitamin D supplementation (>800 IU/day).

### 4.2. Baseline Evaluation and Data Collection

Patient data at baseline were extracted from medical records and included demographic characteristics (age, sex), diabetes type and duration, glycated hemoglobin (HbA1c) values, presence of systemic conditions such as hypertension, dyslipidemia, and diabetic nephropathy, as well as ocular findings including best-corrected visual acuity (BCVA), central macular thickness (CMT), and relevant spectral-domain optical coherence tomography (SD-OCT) indicators.

Serum 25-hydroxyvitamin D [25(OH)D] concentrations were determined using a chemiluminescence immunoassay. Vitamin D status was categorized as follows: deficiency (<20 ng/mL), insufficiency (20–30 ng/mL), and sufficiency (>30 ng/mL) [11].

SD-OCT assessments were conducted at baseline and at 5-week follow-up using the Topcon DRI OCT Triton system. Visual acuity was recorded using standardized ETDRS Early Treatment Diabetic Retinopathy Study charts under controlled conditions.

### 4.3. Treatment Protocol

Participants were administered intravitreal aflibercept at a dose of 2.0 mg/0.05 mL under a pro re nata (PRN) treatment approach. Re-injection decisions were guided by either persistent or recurrent central macular thickness exceeding 300 μm, or a reduction in best-corrected visual acuity. Re-treatments were assessed and administered at five-week intervals based on both clinical judgment and optical coherence tomography OCT findings. All procedures were carried out in sterile conditions within a surgical suite.

### 4.4. Outcome Measures

The primary endpoints of this study were changes in best-corrected visual acuity (expressed in logMAR) and central macular thickness (measured in micrometers) between baseline and the fifth week. Secondary analyses explored the relationship between initial serum 25(OH)D levels and treatment efficacy, which was defined as either an improvement of ≥0.1 logMAR or a ≥10% reduction in central macular thickness. Participants were categorized into vitamin D deficiency, insufficiency, or sufficiency groups according to their serum concentrations

## 5. Conclusions

In summary, this study indicates a possible association between serum vitamin D levels and short-term treatment outcomes following ianti–vascular endothelial growth factor -VEGF therapy in diabetic macular edema. Although the results did not reach statistical significance, likely due to the limited cohort size, consistent trends suggest that vitamin D sufficiency may correlate with more favorable visual and anatomical responses. Further large-scale, controlled studies are required to validate these findings and evaluate the role of vitamin D as a predictive biomarker in personalized retinal disease management.

## Data Availability

Data is contained within the article.

## Abbreviations

25(OH)D: 25-hydroxyvitamin D; AV_f: Final Visual Acuity; AV_i: Initial Visual Acuity; BCVA: Best Corrected Visual Acuity; BMI: Body Mass Index; BRB: Blood–Retinal Barrier; CMT: Central Macular Thickness; CRT: Central Retinal Thickness; DME: Diabetic Macular Edema; DR: Diabetic Retinopathy; ETDRS: Early Treatment Diabetic Retinopathy Study; HbA1c: Glycated Hemoglobin; HDL: High-Density Lipoprotein; IDF: International Diabetes Federation; IU: International Units; LDL: Low-Density Lipoprotein; OCT: Optical Coherence Tomography; ROS: Reactive Oxygen Species; SD-OCT: Spectral-Domain Optical Coherence Tomography; TG: Triglycerides; VEGF: Vascular Endothelial Growth Factor.
